# Editorial: Ultrahigh field metabolic MRI: Current status, clinical applications, and future perspectives

**DOI:** 10.3389/fneur.2023.1119965

**Published:** 2023-01-24

**Authors:** Remy Guillevin, Anja G. van der Kolk

**Affiliations:** ^1^Centre Hospitalier Universitaire (CHU) de Poitiers, Poitiers, France; ^2^LRCOM I3M, Poitiers, France; ^3^UMR7348 Laboratoire de mathématiques et applications, Futuroscope, Chasseneuil-du-Poitou, France; ^4^University of Poitiers, Poitiers, France; ^5^Department of Medical Imaging, Radboudumc, Nijmegen, Netherlands; ^6^Department of Radiology, University Medical Center Utrecht, Utrecht, Netherlands

**Keywords:** ultra high field MRI, multinuclear imaging, metabolic imaging, brain tumors, radiogenetics

Restricted for a long time to fundamental research, ultrahigh field (UHF) MRI has undergone significant development of its clinical applications since 2017. Indeed, the CE/FDA approval obtained for certain 7T magnets has allowed the installation of clinical platforms in several institutions around the world, steadily increasing the number of 7T platforms worldwide. The UHF strength comes with an increased signal-to-noise ratio (SNR) that can be utilized to either achieve a higher spatial resolution within a still-reasonable acquisition time, or alternatively a similar spatial resolution but significantly faster than lower field strengths. This decrease in acquisition time can also be used for faster dynamic sequences, for instance in cardiac imaging. In addition, the increased sensitivity to susceptibility effects together with the high SNR enable clearer delineation of anatomical structures, even at lower spatial resolutions. These advantages have led to several clinical applications, including epilepsy in which a 30% increase in the rate of lesion identification has been found. Despite technical difficulties and imaging artifacts related to the use of UHF (e.g., field inhomogeneities), technological solutions are progressively being developed.

Nevertheless, technical advances at lower field strengths—like acceleration techniques or new AI applications that can synthesize equally high-resolution, high contrast-to-noise ratio (CNR) images from lower field data—have started to make clinically more accepted lower field strengths serious competitors regarding image detail and speed. However, another advantage of UHF MRI that cannot be readily achieved at lower field strengths is its increased spectral resolution and consequent increased sensitivity to (macro) molecules and nuclei other than protons (x-nuclei) that could be of clinical interest. For instance, magnetization transfer techniques at UHF provide more detailed information on different metabolites, which can be utilized to for instance probe the structure of myelin, an essential element in the transport of nerve impulses. Another example is MR spectroscopy, which seems to have never really achieved its full potential in the clinic, not in the least due to overlapping metabolites hampering true tissue probing. UHF MRI therefore could give us access not only to anatomy with a precision that is not readily accessible on standard clinical devices, but also to metabolism, ionic homeostasis, and energetic processes *in vivo* within the human body with an unmatched quality. In addition, its non-invasive character allows repeated measurements and thus the generation of kinetic profiles, opening up the possibility of producing biometabolic models that no longer systematically use animal models. Thus, UHF imaging allows to move from an anatomical representation to a dynamic metabolic representation.

The articles in this Research Topic provide just a glimpse of the full potential of UHF MRI to visualize metabolites and metabolic pathways, using either proton or x-nuclei imaging. Although the latter necessitates new hardware to be developed, the UHF community has ample experience in this field, as shown in the article by Forner et al. who used specially designed coils for phosphorus spectroscopy exploration of tongue tumors. Phosphorus imaging is also used by Korzowski et al. who show the improvement of regional glioma mapping using ^31^P MRS. High spatial resolution sampling allows a better characterization of intra- and peritumoral tissue heterogeneity. This possibility to obtain, without sampling or biopsy, metabolic or genetic information predictive of clinical outcome defines a personalized follow-up in line with 5Ps medicine. Jumping from one x-nucleus to another, Stobbe et al. shows the possibility of identifying and quantifying an alteration of the intra-extracellular sodium compartmentalization in relation to Multiple Sclerosis. Grimaldi et al. even identified new biomarkers using sodium MRI and thus envisaged new pathophysiological approaches in idiopathic Parkinson's disease, by showing an abnormal sodium accumulation within dopaminergic neurons.

It is not only x-nuclei imaging where UHF MRI excels compared to lower field strengths. Quantification—instead of using ratios—of neurotransmitters is now a possibility, for instance glutamate which is studied in two studies presented in this Research Topic. O'Grady et al. uses a CEST sequence to measure glutamate in MS patients, finding a correlation between glutamate in certain regions and disability and cognitive function, while Park et al. developed a protocol for glutamate and GABA measurements that can be used for probing the changes of these neurotransmitters under different conditions, like hyperglycemia. Finally, Shams et al. shows the potential of UHF metabolic MRI to extract genomic information *in vivo*, by detecting 2OH Glutarate (2HG), a metabolic correlate of the IDH mutation quantified in a much more reliable manner than at 3T.

By *in vivo* functional and metabolic sampling of the brain, UHF paves the way to *in vivo* characterization of genomic mutations by means of their metabolic counterparts. Combining this with AI-driven quantitative analysis of the data could ultimately lead to the development of a “digital twin:” rebuilding a lesion within the organ, allowing virtual biopsy as well as therapeutic simulation (e.g., neurosurgical procedures) and treatment monitoring, all in a noninvasive way.

## Author contributions

All authors listed have made a substantial, direct, and intellectual contribution to the work and approved it for publication.

